# Differences in cyclic fatigue lifespan between two different heat treated NiTi endodontic rotary instruments: WaveOne Gold vs EdgeOne Fire

**DOI:** 10.4317/jced.55839

**Published:** 2019-07-01

**Authors:** Gianluca Gambarini, Massimo Galli, Dario Di Nardo, Marco Seracchiani, Orlando Donfrancesco, Luca Testarelli

**Affiliations:** 1 Department of Oral and Maxillo-Facial Sciences, Sapienza University of Rome, Italy

## Abstract

**Background:**

Aim of this study is to investigate the cyclic fatigue resistance of the Gold treated WaveOne Gold and the Firewire treated EdgeOne Fire instruments. The null hypotesis was that there were no differences between the lifespan of Gold treated and FireWire treated instruments when subjected to cyclic fatigue tests.

**Material and Methods:**

40 new NiTi instruments with a length of 25 mm were tested: 20 Wave One Gold Medium (WOG), tip size 35 and variable taper (Dentsply Maillefer, Ballaigues, Switzerland) and 20 Edge One Fire (EOF) (EdgeEndo, Albuquerque, New Mexico) tip size 35 and the same variable taper. A mobile support for the electric handpiece and a stainless-steel block containing the artificial canals were used. The same artificial root canal with a 90 degrees angle of curvature and 2 mm radius of curvature was used for all the tested instruments and the WOG counter-clock wise reciprocating motion with an engaging angle of 150° and a disengaging angle of 30° at 300 rpm, was selected for the test. All instruments were inserted at the same length (18mm) and then rotated in the same reciprocating motion until fracture occurred: the time was stopped as soon as the fracture was visible and video-recorded with a 1/100 sec chronometer. Differences among groups were statistically evaluated with an analysis of variance test ANOVA (significance level was set at *p*<0.05).

**Results:**

Mean values of time to fracture (TtF) for EOF instruments were 28,00 seconds (SD +/- 2,64) and for WOG instruments were 14,67 seconds (SD +/- 2,41). Statistical analysis found significant differences between the TtF of the two instruments (*p*<0,05).

**Conclusions:**

Firewire instruments resulted to be about two times more resistant to cyclic fatigue when compared with identical instruments made with Gold treatment.

** Key words:**Endodontics, NiTi, Waveone Gold, EdgeOne Fire, Cyclic Fatigue.

## Introduction

The introduction of nickel-titanium alloy and the use of mechanical instrumentation in endodontics improved the quality of root canal treatment with less time consuming procedures. Unfortunately, with the use of mechanical devices, the intra-operative fracture of an endodontic instrument has become a more common accident due to the increased torsional and cyclical fatigue stresses (1-3). The application of less stressing movements as such as alternate or reciprocating motion rather than continuous movement, reduced significantly the risk of intracanal leakage of the endodontic instrument (4). The mechanical properties of endodontic instruments have been investigated using different techniques: bending resistance tests and flexural cyclic fatigue resistance tests are usually performed by the use of artificial canals with different curvatures at room or body temperature, due to the impossibility to obtain standardization with natural extracted teeth (5-9).

Static and dynamic tests could be performed to investigate resistance to bending and cyclic fatigue lifespan: dynamic tests are usually similar to the static ones, with the adjunction of an axial movement that simulates a clinical approach (10-12).

Mechanical properties of nickel-titanium endodontic instrumentation like fatigue resistance, flexibility, cutting efficiency and canal centering ability have been improved since the introduction of thermal treatments (13-15). Thermally treated NiTi alloys are characterized by a higher percentage of martensitic phase that is more flexible and resistant to fatigual stresses rather than the austenitic phase. The most common proprietary thermal treated NiTi alloys are known as M-Wire, Gold Technology, Blue Technology (Dentsply, Tulsa, OK, USA) controlled memory wire or CM-Wire (Coltene, Cuyahoga Falls, OH, USA) and R-phase wire (SybronEndo, Orange, CA, USA) (16).

Instead of the M-wire, where the alloy is thermally treated before the manufacturing, instruments subjected to gold treatment technology are heated and then slowly cooled after they have been manufactured (5,17).

Blue treatment produces titanium oxide which gives a typical blue aspect to the surface of the instrument. This treatment allows the instrument to easily reach the martensitic phase during the clinical use instead of other alloys, which are predominantly in the more rigid austenitic phase. It also provides the possibility of softly precurve the instrument like a stainless steel file, and an improved resistance to cyclic fatigue when compared with other thermally treated alloys (18,19).

WaveOne Gold (Dentsply Maillefer, Baillagues, Switzerland) is a new generation Gold treated single-file system for single use procedures: the cross-section of the file is a parallelogram with two cutting edges and the same off-center design used in ProTaper Next (Dentsply Maillefer, Baillagues, Switzerland) (5).

EdgeOne Fire (EdgeEndo, Albuquerque, New Mexico, USA) presents the same cross section of the WOG and it is designed to be used in reciprocating motion, with the same handpiece settings adopted for WaveOne Gold: these endodontic instruments are treated with a proprietary heat process called FireWire™, that it is claimed to provide high flexibility and a negligible restoring force. However, no studies on this type of thermally treated instruments could be found in literature yet.

Aim of this study is to investigate the cyclic fatigue resistance of WaveOne Gold and EdgeOne Fire instruments. The null hypotesis was that there were no differences between the lifespan of Gold treated and FireWire treated instruments when subjected to cyclic fatigue tests.

## Material and Methods

**Figure 1:**
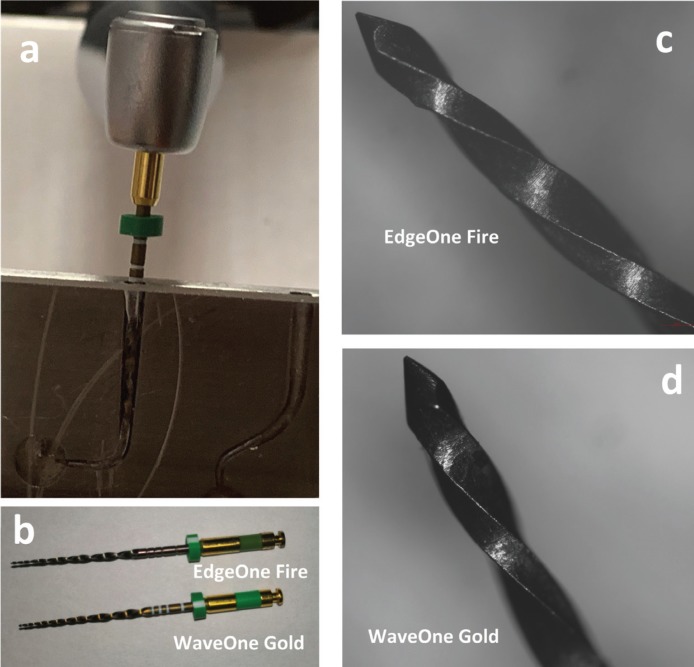
The artificial canal with the inserted instrument. (a) Macrospical (b) and microscopical (20x) aspect of the tested instruments: EdgeOne Fire (c) and WaveOne Endo (d).

40 new NiTi instruments with a length of 25 mm were tested: 20 Wave One Gold Medium, tip size 35 and variable taper (Dentsply Maillefer, Ballaigues, Switzerland) and 20 Edge One Fire (EdgeEndo, Albuquerque, New Mexico) tip size 35 and the same variable taper. Using a stereomiscrope at 20x magnification, the instruments were previously examinated for macroscopical defects or visible signs of deformations: none of them were discarded (Fig. 1).

Both the instruments were used with the same preset program specific for the WaveOne Gold instruments, because Edge One Fire has no preset motion and manufacturer declare its full compatibility with WOG counter-clock wise reciprocating motion with an engaging angle of 150° and a disengaging angle of 30° at 300 rpm.

A cyclic fatigue device already validated in previous studies was used (15,20). The device is made by two parts: a mobile support for the electric handpiece and a stainless-steel block containing the artificial canals. The mobile device ensuring the handpiece allows a precise and reproducible placement of each instrument inside the stainless steel canal, ensuring that each instrument reached the same depth (18 mm) (Fig. 1).

The same artificial root canal with a 90 degrees angle of curvature and 2 mm radius of curvature was used for all the tested instruments. The whole procedure was performed by the same operator, to reduce the variability due to operator skills during the testing procedure.

All instruments were inserted at the same length (18mm) and then rotated in the same reciprocating motion until fracture occurred. For each instrument, the time was stopped as soon as the fracture was visible and video-recorded with a 1/100 sec chronometer. Time to fracture for each instrument was recorded (TtF).

**Figure 2:**
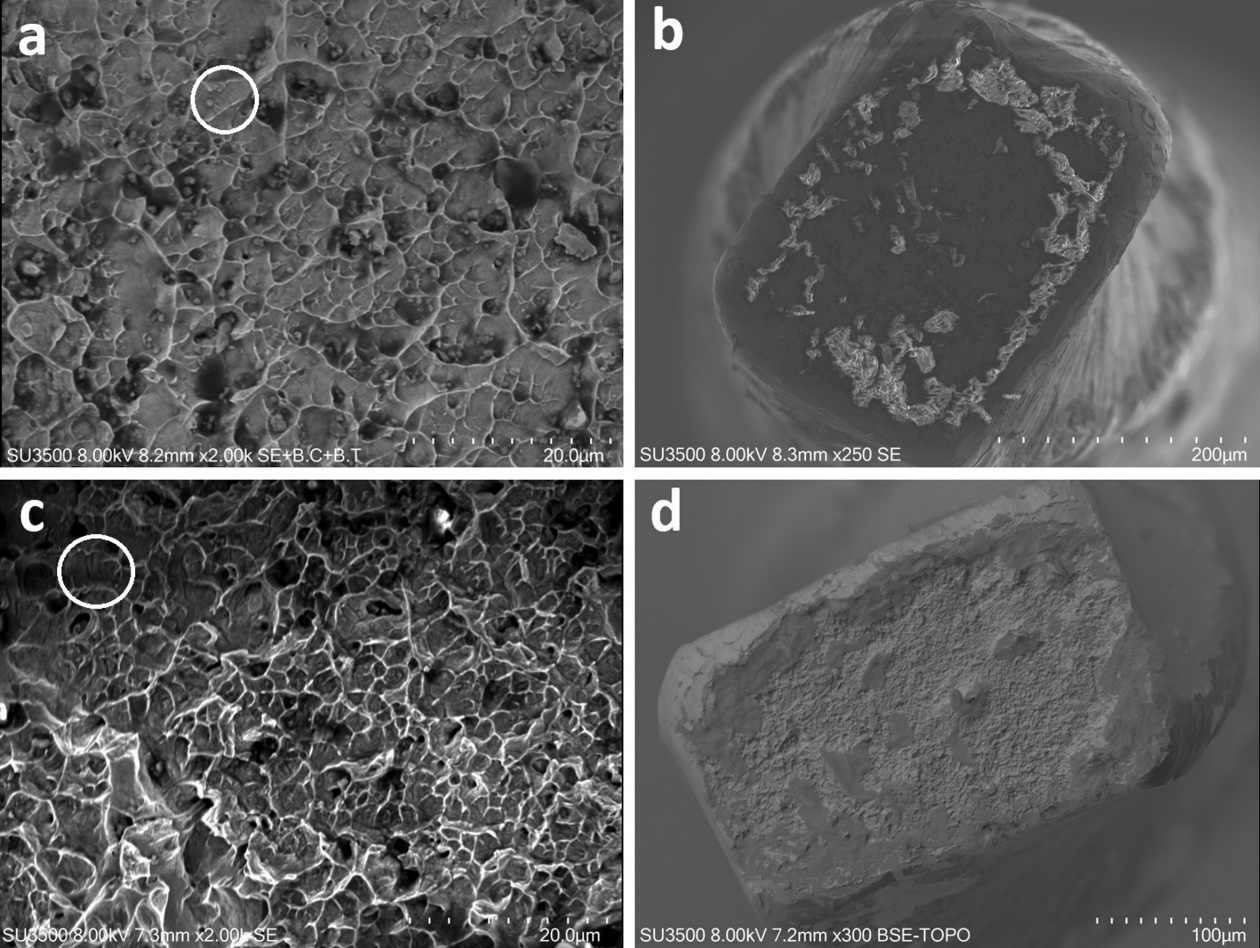
SEM magnification of the fractured instrument: WOG (a-b) and EoF (c-d). White circles show the striations made by cyclic fatigue.

Fragments were collected, measured by using a digital caliber and subject to SEM analysis to ensure that all instruments reached a fatigual separation, demonstrated by the presence of striations on the fractured surface (Fig. 2).

Data were collected and mean and standard deviation were calculated. Differences among groups were statistically evaluated with an analysis of variance test ANOVA (significance level was set at *p*<0.05). Data were statistically analyzed using the SPSS 17.0 software (SPSS Incorporated, Chicago, IL, USA).

## Results

Mean values for fragment length, showing no statistically relevant differences (*p*>0,05), demonstrated that both instruments were subjected to the stresses in the same portion.

Mean values of TtF for EOF instruments were 28,00 seconds (SD +/- 2,64) and for WOG instruments were 14,67 seconds (SD +/- 2,41) ( Table 1). Statistical analysis found significant differences between the TtF of the two instruments (*p*<0,05), so the null hypotesis was rejected.

**Table 1:**
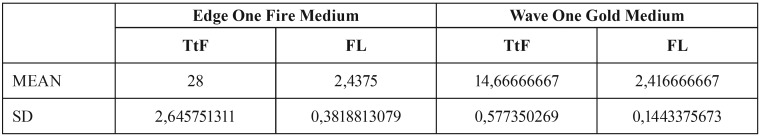
Instruments’ mean time to fracture (TtF) and fractured segments’ length (FL).

## Discussion

To optimally analyze the properties of a heat treatment, it should be used the same designed instruments at the same conditions due to remove bias caused by different volumes of the alloy, cross-sectional shape and cutting performance (21,23).

Cross-sectional design and type of mechanical movement are considered crucial factors in the affection of cyclic fatigue resistance of an endodontic instrument (24,25).

The use of artificial canals seemed to be useful to eliminate differences between teeth like dentine hardness, canal length and curvature degree and radius: standardization of the canal is necessary to avoid unexpected interferences which could invalidate the tests (26,27). In this study, a very hard curvature (90°) has been selected due to evaluate the instrument’s behavior in critical conditions. In the clinical practice, it’s rare to find so hard curvatures, but it was expected that easier curvatures will result in longer time to fracture and for this reason, they were discarded.

In literature, heat treated NiTi alloys demonstrated improvements in mechanical properties like cyclic fatigue resistance which could extend the instrument’s lifespan when compared with no heat treated or different heat treated instruments (28,29).

WaveOne Gold are made by Gold-wire NiTi, which is considered a heat treatment that could modify the metallurgic behavior, increasing the flexibility of the instrument. To evaluate the effectiveness of Gold-wire instruments, studies used to perform cyclic fatigue tests by using same shaped instruments, treated with different thermal procedures. Results indicate that thermal treatments improved flexibility and cyclic fatigue lifespan of the endodontic instruments when compared with non treated instruments (30-32).

In this study, the tested instruments, EdgeOne Fire and WaveOne Gold, have the same three-dimensional characteristics: they are similar in design and they should be used with the same reciprocating program, as suggested by the producer. To date, no studies on EdgeOne Fire instruments have been published yet.

When all the controllable variables or external influences have been standardized or removed to reduce bias, results evidenced the superiority in terms of cyclic fatigue lifespan of the EdgeOne Fire when rotated at the same speed, in the same canal (90° curvature and 2 mm radius) with the same reciprocating motion.

Actually, Gold and Blue treated instruments represent the newest technology in the field of endodontic instruments: they have been tested in many articles, and results reported relevant differences in terms of cyclic resistance. Blue treated instruments resulted to be more resistant than gold treated ones in many cases (18,29,33).

In this study, Firewire instruments resulted to be two times more resistant to cyclic fatigue when compared with identical instruments made with Gold treatment. This result could be due to the peculiar three-dimensional aspect of the cristalline matrix of the Fire-wire alloy which confers an higher flexibility and cyclic fatigue resistance to the instruments (34).

## Conclusions

EdgeOne Fire instruments, made with a proprietary thermal “Fire-wire” treatment, showed an improved resistance to cyclic fatigue when compared with similar instruments made with Gold-wire technology. Further studies should be aimed to compare the Fire-wire technology with the latest technologies in terms of thermal treatments, to analyze and assess if there are differences in flexibility and cyclic fatigue resistance between these alloys.
